# A two-stage deep-learning framework for image classification of 46 southeast Asian fruit and vegetable categories

**DOI:** 10.3389/fpubh.2026.1875869

**Published:** 2026-08-25

**Authors:** Decho Surangsrirat, Panyawut Sri-iesaranusorn, Warisara Asawaponwiput, Wassapon Watanakeesuntorn, Polathep Vichitkunakorn

**Affiliations:** 1 Digital Healthcare Platform Innovation Group, National Science and Technology Development Agency, Pathum Thani, Thailand; 2 Canterbury Technical Institute, Brisbane, QLD, Australia; 3 D3 Center, Osaka University, Osaka, Japan; 4 Department of Family and Preventive Medicine, Faculty of Medicine, Prince of Songkla University, Songkla, Thailand; 5 Health Policy Research Center, Faculty of Medicine, Prince of Songkla University, Songkhla, Thailand; 6 Research and Innovation Center for Well-being and Continuing Care, Prince of Songkla University, Hat Yai, Songkhla, Thailand

**Keywords:** deep learning, food image classification, fruit and vegetable classification, mHealth, semi-supervised learning

## Abstract

**Introduction:**

The ThaiSook mHealth application was designed to promote a healthy lifestyle and mitigate risk factors associated with noncommunicable diseases; one of the administrative tasks involved is the manual flagging and reporting of incorrect entries related to fruit and vegetable intake. However, the process can be time-consuming and prone to errors. Automating food categorisation can provide immediate insights into dietary choices and encourage healthier eating habits. Therefore, we aimed to develop a food image classification model that can be used to categorise different types of Southeast Asian fruits and vegetables, for which nutritional data are available from the Thailand Ministry of Public Health, using the ThaiSook mHealth application.

**Methods:**

A two-stage model encompassing binary and multiclass classification stages was developed to classify 46 types of Southeast Asian fruit, vegetables, and vegetable-heavy dishes. We used a two-stage strategy featuring binary and multiclass models; EfficientNetV2S emerged as the optimal choice for the classification model. The binary classification phase eliminated non-fruit and non-vegetable images, whereas the subsequent multiclass classification identified specific fruit or vegetable types. The methodology incorporates semi-supervised learning using Noisy Student training, complemented by techniques such as pseudo-labelling, label smoothing, and temperature scaling. These strategies enhance model performance and robustness and effectively exploit unlabelled data.

**Results:**

The dataset included the labelled group (5,760 fruit and vegetable images and 5,450 non-fruit or vegetable images) and unlabelled group (29,385 images; contributed by ThaiSook application users). Our system’s comprehensive performance showed that the end-to-end pipeline achieved accuracies of 92.43, 94.56, and 94.91% in the top-1, −3, and −5 experiments, respectively.

**Conclusion:**

We developed a robust image classification model tailored to fruits and vegetables that can be practically applied for dietary assessment using the ThaiSook mHealth application. Our findings demonstrate the resilience and suitability of our model for resource-constrained mobile applications.

**Trial registration:**

The clinical trial was registered according to the WHO International Clinical Trials Registry Platform (WHO-ICTRP) in the Thai Clinical Trials Registry (ID TCTR20220611001) on 11 June 2022.

## 1 Introduction

Recently, the use of mobile health (mHealth) technology has increased remarkably worldwide, reflecting a transformative shift in the healthcare delivery landscape. Statistically, the market for the global mHealth app has exhibited substantial growth, with an anticipated compound annual growth rate of 25.3% between 2021 and 2022 (1). Notably, the Asia–Pacific region has become the epicentre of the mHealth revolution, with the fastest-growing market for mHealth apps (2–4). Thailand stands out as a prime example of remarkable growth in mHealth app adoption, with 66.8% of the general population actively embracing health and wellness apps in 2020. Furthermore, diet and nutrition logging have become prominent and widely utilised features among the array of functions offered by these apps.

With the advance in technology, there has been considerable progress regarding accuracy and efficiency in food-image classification. These improvements in accuracy enable individuals to make more informed decisions regarding their dietary preferences. Automated nutritional analysis driven by advanced food image classification technology provides users with a convenient and accurate method for tracking their diet. Individuals can gain immediate insight into their nutritional choices by instantly recognising and categorising foods from images, thereby promoting more informed and balanced eating habits. Moreover, the efficiency of these systems has improved, allowing for real-time recognition and food analysis, and reducing the time and effort required for manual data entry or analysis significantly.

Regarding food image classification, Islam et al. (5) harnessed the capabilities of a pre-trained Inception V3 model to effectively categorise images within the food-11 dataset. Furthermore, Yadav et al. (6) introduced data augmentation techniques and leveraged the visual geometry group-16 architecture to achieve a reasonable accuracy of 85.07%. Regarding fruit and vegetable classification, Ahmed et al. (7) investigated the use of a convolutional neural network (CNN)-based model on a dataset comprising 15 distinct categories of vegetable images comprehensively, which resulted in a substantial corpus of 21,000 images. Sumanth et al. (8) employed MobileNetV2, a lightweight deep learning model that provides a balance between classification accuracy and computational efficiency, making it particularly suitable for mobile and embedded vision applications. In addition, Gill and Khehra (9) introduced an integrated model, in which CNNs, recurrent neural networks, and long short-term memory components were combined for fruit image classification. Their model outperformed conventional classifiers, including the Adaptive Neuro-Fuzzy Inference System, underscoring the effectiveness of deep neural networks in the intricate task of fruit image classification. In the domain of transformer-based models, Rusheek et al. (10) proposed a Vision Transformer (ViT) for food image classification, whereas Xiao et al. (11) adopted the Swin Transformer architecture. While these models demonstrate superior classification performance, they require greater computational resources and exhibit longer inference times, highlighting the trade-off between accuracy and efficiency. The MobileNet family of models is designed for efficient mobile and embedded vision applications (12). Introduced in 2019, MobileNetV3-Small is optimised for resource-constrained environments (13). Owing to their lightweight architecture, computational efficiency, and demonstrated performance across various applications (14–16), models of this family are strong candidates for mobile image classification. In this study, several lightweight architectures, including MobileNetV3-Small, ConvNeXt-Tiny, and EfficientNetV2S, were empirically compared, and the best-performing model was selected as the backbone (Section 3).

The findings from these studies highlight the achievements in their respective datasets. However, recognising that these studies were primarily tailored for tasks involving the categorisation of foods, fruits, and vegetables is crucial. Some of these methods may not be optimal for more advanced food classification, particularly for food logging in mobile applications. In such instances, using recommendation systems wherein the results are ranked could provide a better solution instead of relying solely on model-generated outputs.

The ThaiSook mHealth application, developed by the National Science and Technology Development Agency (NSTDA), was designed to promote a healthy lifestyle and mitigate risk factors associated with noncommunicable diseases (NCDs). These outcomes were achieved through a virtual competition model. The model was designed to incorporate expert coaching, foster team dynamics, and encourage friendly competition among groups, all with the prospect of winning rewards. The application operates as a user-driven programme that allows individuals to self-record key predictors of premature mortality, including weight, vegetable and fruit intakes, physical activity levels, blood pressure, blood lipid profiles, blood glucose levels, and smoking habits. These seven parameters are closely related to the principles outlined in Folsom’s publication, *American Heart Association’s Life’s Simple 7: Avoiding Heart Failure and Preserving Cardiac Structure and Function* (17). These predictors are strongly correlated with premature mortality (17, 18), and ThaiSook tracks and records these variables. Individuals can monitor and improve their health with its user-friendly interface, aligning with the overarching mission of the application to enhance their well-being and reduce the burden of noncommunicable diseases.

Automating food categorisation from images provides users with immediate insight into their dietary selection, motivating them to adopt more informed and well-balanced eating habits. As the ThaiSook mHealth application promotes healthy lifestyles through virtual competition, one of the administrative tasks involved is the manual flagging and reporting of incorrect entries related to fruit and vegetable intake. Considering the substantial volume of images in each competition, the process can be time-consuming and prone to errors. The automation of this task significantly reduces the workload, screens out errors, and saves time. In addition, it promotes user engagement and learning by allowing individuals to recognise and rectify mistakes.

**Figure 1:**
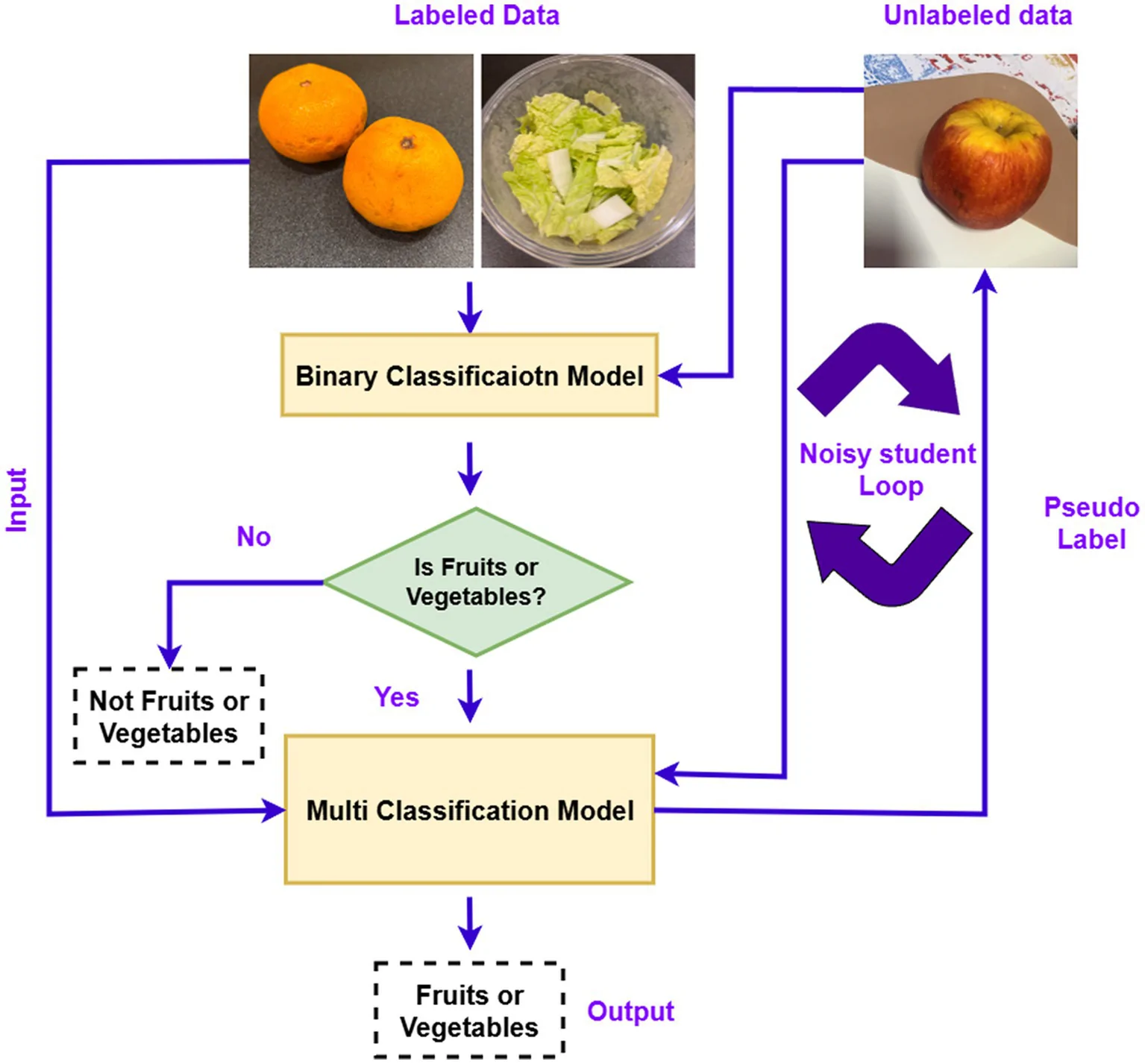
Overview of the classification process performed in this study. The model consists of a binary classification model to remove non-fruit or non-vegetable images, followed by a multi-class classification model to categorise the specific type of fruit or vegetable.

Therefore, we aimed to develop a food image classification model focusing on fruits and vegetables, which are the cornerstones of a healthy diet. Partnering with the mHealth ThaiSook application’s team, our experiment mainly focused on developing a machine-learning model (Figure 1) that can be used to categorise 43 different types of Southeast Asian fruits and vegetables for which nutritional data are available from the Thailand Ministry of Public Health. Furthermore, we included three classes of vegetable-rich dishes: papaya salads (Somtam), stir-fried vegetables, and vegetable salads.

To address challenges related to limited labelled data and diverse food appearances, we propose a two-stage semi-supervised framework that integrates binary classification, multiclass recognition, pseudo-label generation, and Noisy Student training to leverage unlabeled data. Additionally, label smoothing and temperature scaling are incorporated to improve prediction reliability and robustness for practical food image recognition.

## 2 Materials and methods

Our model comprised a binary classification stage, followed by a multiclass classification stage (Figure 1). A binary classification was used initially to filter out non-fruit or non-vegetable images. Subsequently, a multiclass classification model was applied to categorise specific types of fruits or vegetables.

To improve the performance and robustness of the model and use unlabelled data, we employed various techniques, including pseudo-labelling, label smoothing, temperature scaling, and self-training. The following subsections provide a detailed overview of the methodology, which entails the training process of the baseline model, strategies for mitigating overconfidence, and semi-supervised learning.

### 2.1 Dataset

**Figure 2:**
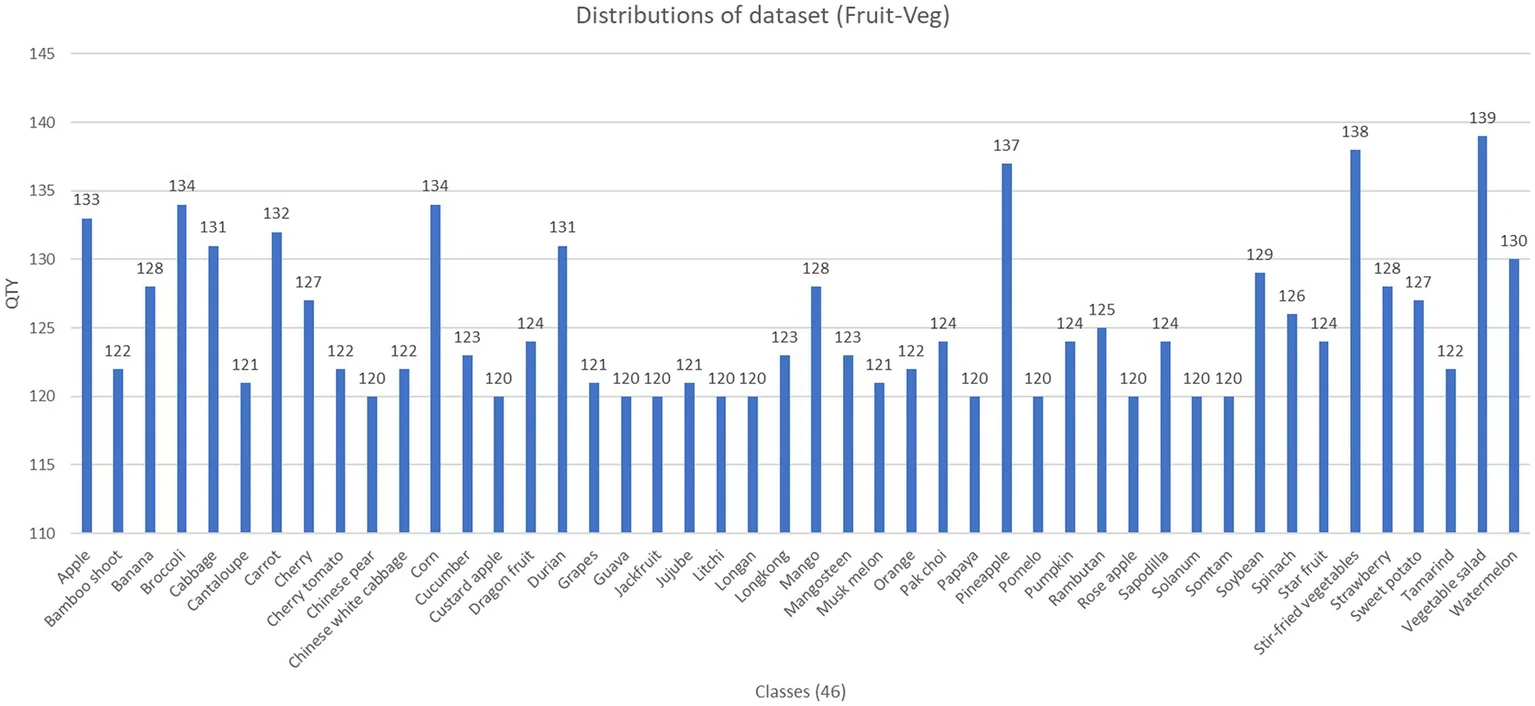
Distribution of images across the 46 classes in the Fruit-Veg dataset.

**Figure 3:**
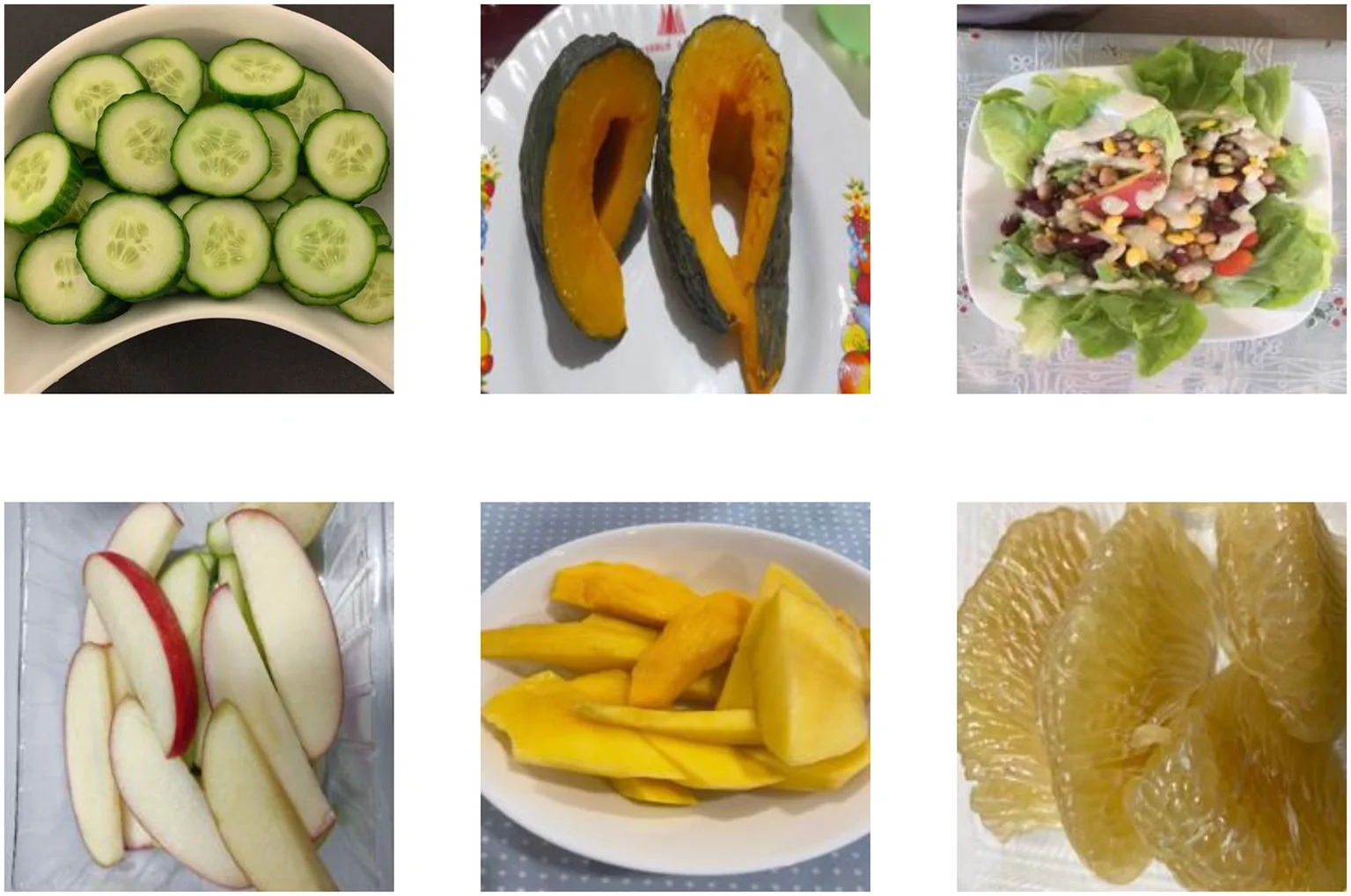
Unlabelled vegetable and fruit images collected from users of the ThaiSook mobile application.

The dataset comprised 40,595 images from ThaiSook database, consisting of Lu et al. (1) a labelled group comprising 5,760 images of fruits and vegetables sourced from verified databases and 5,450 images that did not contain fruits or vegetables (others). The overall dataset distribution between the fruit and vegetable category and the other category is reported in Table 1, including the number of classes and images in each category. The class-wise distribution of fruit and vegetable images is presented in Figure 2 to provide further details on the dataset composition and (2) an unlabelled group comprising 29,385 images contributed by users of the ThaiSook application, as the application allows users to upload images of their meals to track their dietary habits. We obtained the unlabelled images from the application at the beginning of our experiments. The images in this dataset represent 43 distinct types of Southeast Asian fruits and vegetables, for which their nutritional data are accessible through the Thailand Ministry of Public Health (19). Furthermore, three categories of vegetable-rich dishes were included in the dataset: papaya salads (Somtam), stir-fried vegetables, and vegetable salads. Figure 3 shows images of unlabelled vegetables and fruit images gathered from users of the ThaiSook mobile application. All labelled images were manually reviewed and annotated by the authors according to the predefined food categories, with each image assigned to its corresponding fruit, vegetable, or ‘Others’ class based on its visual content. Before training, all collected images were manually inspected to remove duplicate, corrupted, irrelevant, or incorrectly labelled samples, and images of poor visual quality were excluded; ambiguous samples identified during verification were corrected or removed. As each fruit and vegetable class contains approximately 120–139 images, the class distribution is relatively balanced, and no additional balancing techniques, such as oversampling or class weighting, were required.

**Table 1:** Summary of dataset distribution across categories.

Dataset	Classes	Quantities
Fruits and Vegetables	46	5,760
Others	1	5,450
Total	47	11,210

The training dataset was partitioned into a training and test set in an 8:2 ratio. The images were resized to 224 × 224 pixels before they were fed as the input. Cross-entropy was the loss function employed by the model because it can quantify the difference between the probability distribution of the ground truth and the predicted likelihood. The Adam optimizer was selected for optimization, and all its parameters were configured based on the recommendations outlined in the reference paper (20) (learning rate = 0.001, *β*₁ = 0.9, β₂ = 0.999, and *ε* = $1\times 10^{-7}$ ). A batch size of 64 was used for all experiments. Training was performed for a maximum of 100 epochs, with early stopping applied to prevent overfitting. Specifically, training was terminated when the validation loss failed to decrease by more than 0.01 from the current minimum for 10 consecutive epochs, and the model with the best validation performance was retained for evaluation. All experiments were conducted using TensorFlow (version 2.20.0) with Python 3.12.13 on Google Colab running Linux, equipped with an NVIDIA Tesla T4 GPU and 12.7 GB RAM.

### 2.2 Two-stage classification framework

The ThaiSook application operates under the constraints of mobile devices and faces resource limitations that necessitated the development of a classification model tailored specifically for such platforms. The EfficientNet architecture was introduced in 2019 and uses compound scaling to improve classification accuracy while maintaining computational efficiency (21). Shah et al. evaluated EfficientNet-B1 on the Food-101 dataset and found that it outperformed the baseline CNN model, achieving higher classification accuracy while requiring less time for both training and inference (22). For fruit recognition tasks, Duong et al. employed EfficientNet and MixNet to develop an expert system that accurately and efficiently identifies fruits (23).

The use of a two-stage cascaded approach, where one model’s outputs are vital prerequisites for the next model, has been applied to various convolutional neural network scenarios (24, 25). In the context of our application, images without fruits or vegetables should be discarded before they are processed using a multiclass classification model. This precaution was important as the model was not exposed to such images during its training phase. Therefore, including them could potentially lead to erroneous model outputs. We introduced a binary classification model as a precursor to the multiclass classification model in our theoretical framework to significantly reduce the possibility of processing images that lack fruits or vegetables as inputs.

The first stage of our two-stage pipeline was a binary classification model. The model had two classes: ‘fruit-or-vegetable’, comprising 5,760 images of fruits and vegetables, and ‘non-fruit-or-vegetable’, with 5,450 random images that did not contain fruits or vegetables. In the model, an image was forwarded to the next stage of classification when there was high confidence that the image contained a fruit or vegetable, surpassing a specific probability threshold.

### 2.3 Pseudo-labelling and confidence-reducing methods

For binary classification, labelled images were exclusively used in our training process, leaving an extensive unlabelled dataset untapped. This presented a valuable opportunity to enhance the performance of our model by incorporating advanced techniques such as pseudo-labelling, label smoothing, and temperature scaling.

Before performing the semi-supervised learning approach, implementing a pseudo-labelling method is the first essential step (26). This method leverages a model trained on a labelled dataset to predict the labels of an unlabelled dataset. We employed a stringent criterion by setting a high probability threshold, where an unlabelled image received a label assignment only when the highest predicted probability exceeded this threshold. To ensure the quality of pseudo-labels and control the number of labels assigned, scores such as 0.95 were used frequently to predict high-quality pseudo-labels and maintain the number of labels predicted (27–30).

In many instances, the model exhibited overconfidence and made incorrect predictions, particularly when encountering images that differed substantially from those in the training data. Ideally, the model should be more cautious and less confident when encountering unfamiliar images; that is, the probability distribution of the image should be uniform. To address the issue of overconfidence, we explored the following methods.

Label smoothing is a regularisation technique applied to the classifier layer in machine-learning models by estimating the marginalised effect of label dropout during training (31). This method is widely recognised and is used as a fundamental approach for various classification tasks (32, 33). In contrast, the conventional encoding technique, often referred to as ‘hard labels’, assigns a value of 1 to the target class and 0 to all non-target classes. The ‘hard labels’ approach can effectively minimise loss and maximise the likelihood of the target class; however, it inadvertently magnifies the differences between the likelihood of the target class and that of all other classes. Consequently, the model may exhibit excessive confidence when unfamiliar or unknown data are encountered.

Using ‘soft labels’ mitigates the above-mentioned differences by introducing a softer approach to ground-truth values. This adjustment involves introducing two parameters: n, representing the total number of classes, and *α*, denoting the ‘smoothing value’. With ‘soft labels’, the label value assigned to the target class is modified to (1 − α) + $\frac{\alpha }{n}$ , whereas the values for all other classes are set to $\frac{\alpha }{n}$ . This alteration in the ground-truth values directly impacts the weights employed in computing the loss function.

To formalise this concept mathematically, let s_i_ and gt_i_ be the output from the last activation function and the ground-truth value of the i^th^ class, respectively, and let n be the total number of classes. The loss function equation for the model is expressed as follows:


$$\text{Cross}-\text{Entropy}=\sum_{i=1}^{n}gt_{i}*\log(s_{i})$$

Here,


$$gt_{i}=\{\begin{array}{c} (1-\alpha )+\frac{\alpha }{n},\text{The}\;i^{\mathrm{th}}\;\text{class is the target class}\\ \frac{\alpha }{n},\text{otherwise} \end{array}$$

As illustrated in the equation, the loss tends to be greater than that of the ‘hard labels’ approach, which corresponds to the case where *α* = 0. This increased loss induces substantial weight adjustments during backpropagation. Consequently, this method is a regularisation technique that enhances the model’s adaptability to previously unseen data. Based on prior research, we opted for α = 0.10 as the optimal parameter in our experiment (31).

In certain classification scenarios, the probability distributions tend to skew significantly towards extreme values of 0 or 1, indicating that the model exhibits excessive confidence in its predictions. Temperature scaling (34) offers a straightforward solution to this issue. This concept is built on one of the simplest extensions of Platt scaling (35). In temperature scaling, a single scalar parameter is used, denoted by T > 0, where T represents the ‘temperature’. This parameter is used to rescale the logit scores before applying the softmax function, which effectively softens the softmax outputs. In scenarios of overconfidence, we used T > 1 to recalibrate the distribution. The differences between the values were reduced by the recalibration process, making them more evenly distributed than the original values. Notably, because modifying T does not alter the order of the maximum values within the softmax function, class predictions were not affected. Therefore, in theory, it does not affect the accuracy. In previous studies, the T values ranged from 1 to 5 (36, 37). Consequently, in this experiment, we employed a range of T values extending from 1.25 to 2.25, with increments of 0.25. Subsequently, the best-performing model proceeded to the self-training phase.

### 2.4 Semi-supervised learning

Regarding self-training, we implemented a Noisy Student training algorithm with slight modifications (38). An overview of the training procedure is provided in Algorithm 1. Initially, we trained our original model using the labelled images. Subsequently, we used this model, referred to as the ‘teacher’ model, to generate pseudo-labelled images through the previously mentioned pseudo-labelling method. Subsequently, we used the teacher model as a ‘student’ model and trained it using both clean labelled and noisy pseudo-labelled images. This process was repeated iteratively, and a new teacher model based on a trained student was introduced with each iteration. The experimental models involved in this phase included base and reduced-confidence models. In our experiments, we subjected the selected models to a self-training process for five iterations to assess the improvements achieved by using this algorithm.

ALGORITHM 1

**Figure:**
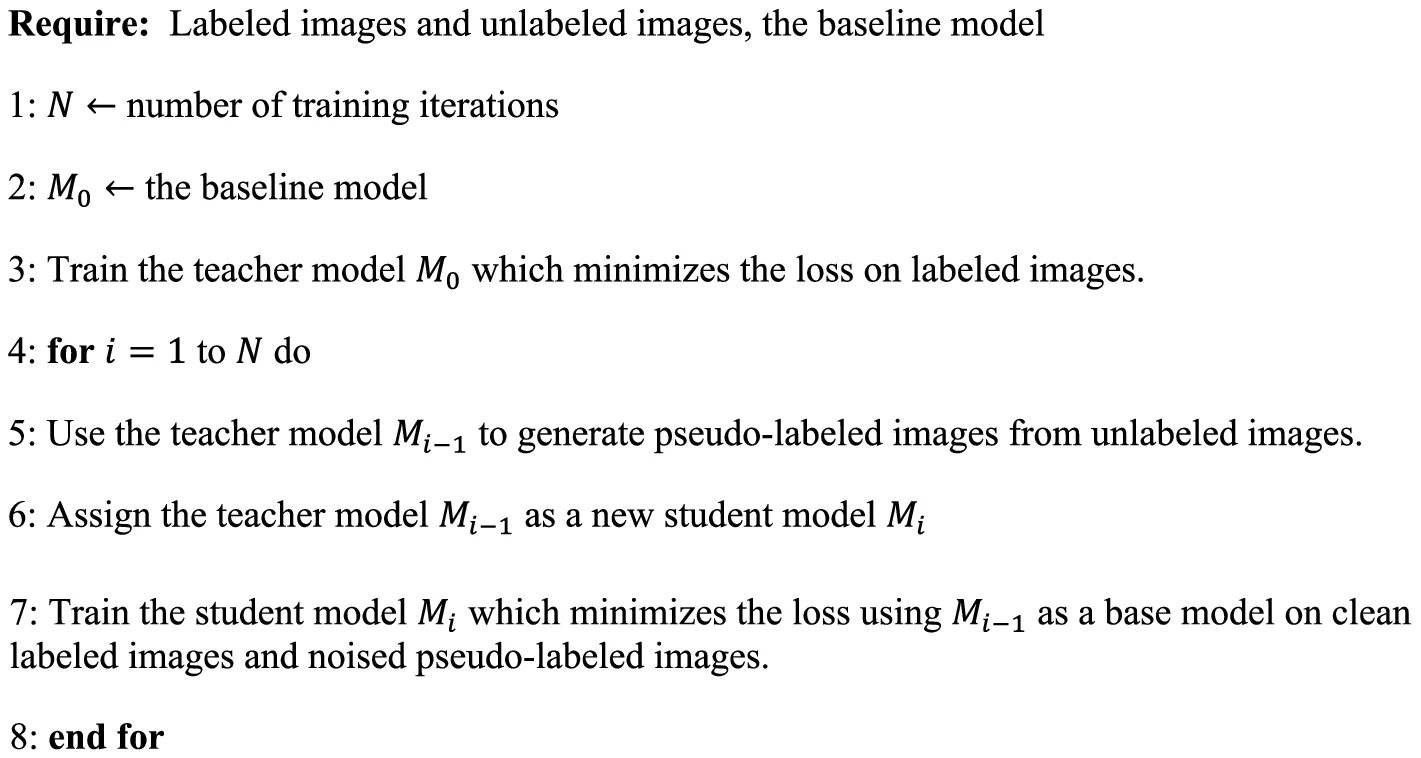
Noisy student training.

**Figure 4:**
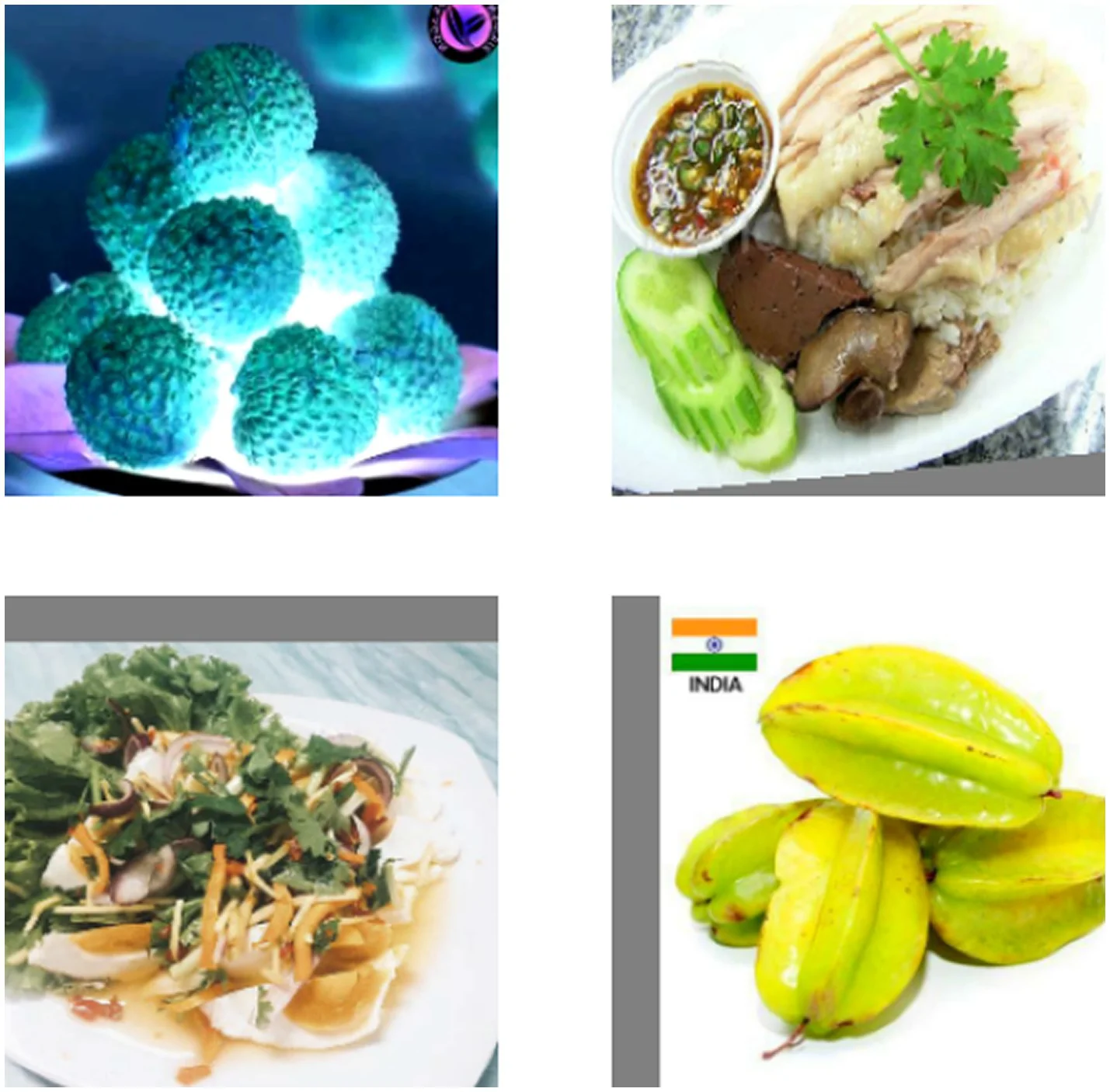
Examples of augmented images using RandAugment function with *N* = 2 and *M* = 9.

Noising strategy is used to mitigate overfitting in the models. In our approach, we introduced noise into the pseudo-labelled images using a simpler strategy than the original Noisy Student training method (38). There were two components in our strategy. First, we applied a 50% dropout rate to the hidden units. Second, we incorporated random augmentation as part of the data augmentation process (39, 40). In the RandAugment function, two parameters, N and M, were used: N is the number of random transformations used in the function and M denotes the transformation strength. As depicted in Figure 4, the parameters N = 2 and M = 9 were adopted in our model, as recommended in previous ImageNet research (40).

### 2.5 Testing and evaluation

In the test protocol, two distinct image sets were used. The first set corresponded to the test split extracted from the labelled dataset, comprising 1,168 images. Concurrently, the second set comprised ‘non-fruit-or-vegetable’ images, comprising 1,091 images. A single parameter, denoted as the ‘fruit-or-vegetables threshold’, was used as a critical factor in the binary model during testing. The evaluation criteria for the test comprised binary accuracy and multiclass top-1, top-3, and top-5 accuracy, which served as key performance indicators. In this study, ‘multiclass top-1 accuracy’ referred to the accuracy of correctly identifying the single most likely class for a given image out of all possible classes. For ‘multi-class top-5 accuracy’, we measured the accuracy of identifying the correct class among the top 5 most likely classes for an image. Top-3 accuracy was defined analogously using the top three predicted classes. These metrics provided insights into the ability of a model to classify images across multiple categories.

In each test iteration, the architecture was tested separately using two image sets. For the ‘non-fruit or vegetable’ set, an image was excluded and recorded as a binary error when it passed the binary classification phase, whereas a non-passing image was categorised as a binary hit. Conversely, for the ‘fruit-or-vegetable’ set, an image discarded after the binary classification phase was counted as a top-1, −3, and −5 error, whereas a passing image proceeded to the multi-classification phase. Nine rounds were included in the testing procedure, during which, the ‘fruit-or-vegetables threshold’ varied. This threshold spanned 0.1–0.9, increasing by 0.1 in each successive round.

### 2.6 Evaluation using a public dataset: Kaggle Fruits-360

To enhance the reliability of our proposed method, we evaluated it using the Fruits-360 dataset available on Kaggle (41), which has been used extensively in previous research (42). This dataset comprises 131 classes of fruit and vegetable images, totalling 90,483 samples. For our evaluation, we randomly selected 100 samples per class for training and retained the original test set from Kaggle.

## 3 Results

We conducted experiments using state-of-the-art models to determine the most suitable model architecture for our experiments. For binary classification, Table 2 presents the results of the seven different models. These results showed that EfficientNetV2S was the most efficient model, with its accuracy surpassing that of the other models, with relative improvements of 45.56, 31.69, 11.22, 9.98, 6.46, and 3.90% compared with those of ResNet50, MobileNetV2, VGGNet-16, VGGNet-19, ConvNext-Tiny, and MobileNetV3Small, respectively. Additionally, EfficientNetV2S exhibited superior precision, recall, and F1-score compared with all other baseline models when a threshold of 0.5 was used. Regarding the area under the receiver operating characteristic curve (AUROC), EfficientNetV2S maintained its lead and outperformed the ResNet50, MobileNetV2, VGGNet-16, VGGNet-19, ConvNext-Tiny, and MobileNetV3Small models by 44.56, 31.19, 11.14, 9.77, 6.14, and 3.84%, respectively. These findings indicate the robustness and effectiveness of EfficientNetV2S.

**Table 2:** Comparison of binary classification results obtained from the seven different models.

Models	Accuracy	Precision	Recall	F1-score	AUROC	PRAUC
ResNet50V2	65.69	67.22	65.69	65.23	66.11	75.07
MobileNetV2	72.73	72.98	72.73	72.72	72.85	78.85
VGGNet-16	85.97	86.00	85.97	85.97	85.99	88.87
VGGNet-19	86.94	87.20	86.94	86.94	87.06	89.43
ConvNext-Tiny	89.82	89.94	89.82	89.83	90.04	92.21
MobileNetV3Small	92.03	92.04	92.03	92.03	92.04	93.66
EfficientNetV2S	95.62	95.67	95.62	95.61	95.57	96.82

AUROC, area under the receiver operating characteristic curve; PRAUC, precision-recall area under the curve.

We identified EfficientNetV2S as the most suitable model architecture for our two-stage pipeline. In this pipeline, the first model was designed to perform binary classification, distinguishing vegetable and food images from the others. Meanwhile, the second model was tailored for categorical classification, aimed at identifying a specific category or type of vegetable or food. To further evaluate the practical applicability of the proposed framework, deployment-related performance metrics were measured for the final model. The proposed model has a model size of 83.16 MB, with an average inference time of 0.1131 s per image, corresponding to 8.84 FPS. The average RAM usage during inference was 1.83 GB. These results provide an assessment of the computational efficiency of the proposed framework and demonstrate its potential suitability for deployment in resource-constrained environments. Although these measurements were obtained in a server-grade environment, the model was deliberately kept compact so that it remains deployable client-side on modern smartphones when required. Conversion to TensorFlow Lite with post-training quantisation, which typically reduces the model size by approximately fourfold, together with on-device latency benchmarking, is planned as future work.

**Figure 5:**
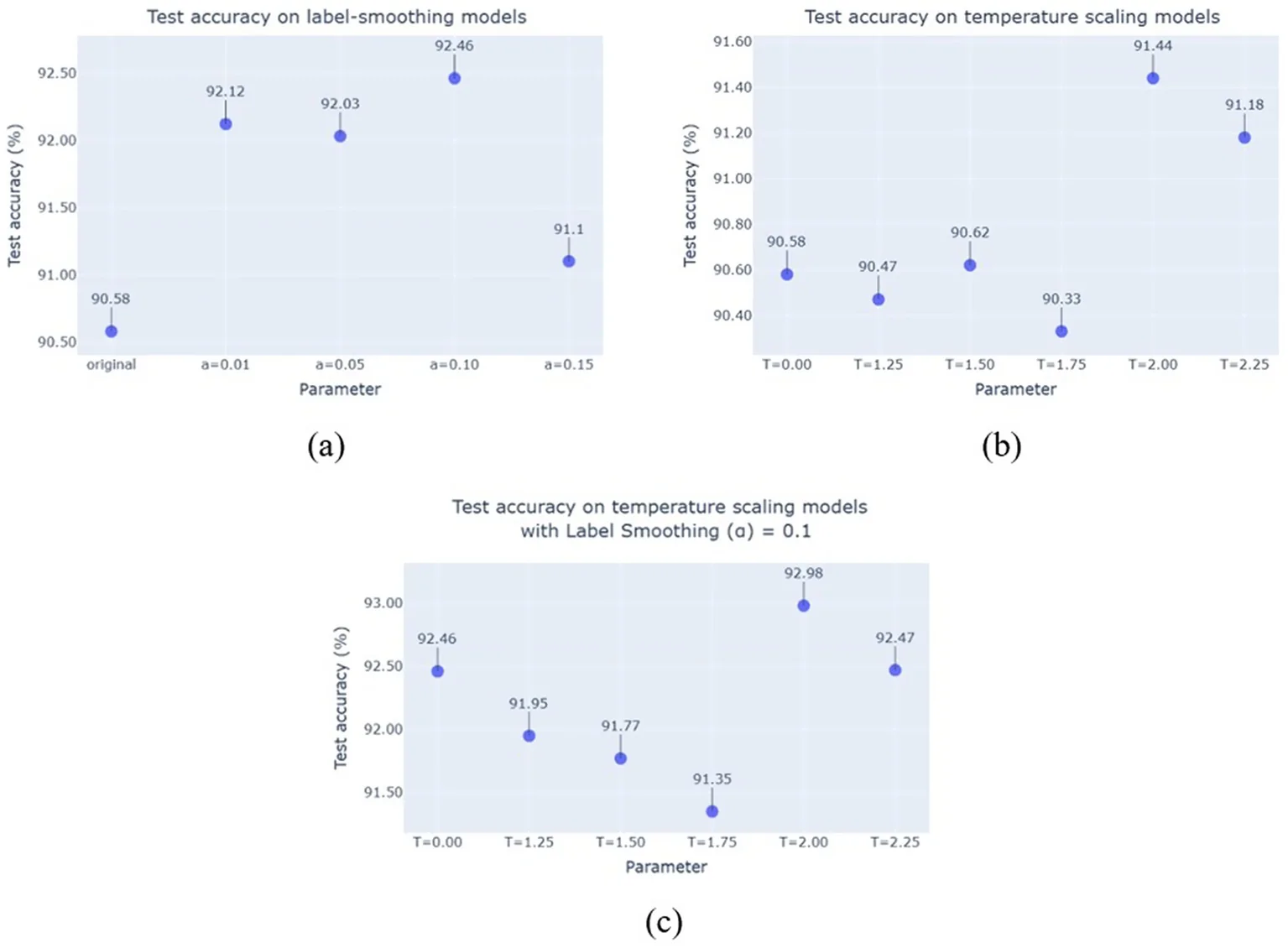
Effects of label smoothing and temperature scaling parameters on categorical classification performance. **(a)** Performance comparison under different label smoothing parameters. **(b)** Effect of temperature scaling without label smoothing (*α* = 0). **(c)** Effect of temperature scaling with the selected label smoothing parameter (*α* = 0.10).

To identify the optimal parameters for label smoothing and temperature scaling, we conducted experiments using various parameters, focusing on their impact on the categorical classification model (Table 3). Figure 5 shows the results for various label smoothing and temperature scaling parameters. In the case of label smoothing, we explored *α* parameters from 0 (original) to 0.01, 0.05, 0.10, and 0.15 (Figure 5a). Subsequently, temperature scaling was investigated using T parameters ranging from 0 (no temperature scaling) to 1.25, 1.50, 1.75, 2.00, and 2.25 under different label smoothing settings. Figure 5b presents the effect of temperature scaling without label smoothing (α = 0), while Figure 5c illustrates the effect of temperature scaling with the selected label smoothing parameter (α = 0.10). The experimental results indicate that the most effective parameters were 0.10 for α in label smoothing and 2.00 for the T parameter in temperature scaling. These chosen parameters demonstrate the contribution of label smoothing and temperature scaling to optimise the performance of our categorical classification model.

**Table 3:** Comparison of results from the semi-supervised Noisy Student training algorithm with various parameters for noising, label smoothing, and temperature scaling on the categorical classification model.

Parameter configuration	Accuracy	Precision	Recall	F1-score	AUROC	PRAUC
Original	90.58	90.89	90.58	90.46	96.50	99.76
α = 0.10, T = 0	92.47	92.70	92.47	92.45	96.74	99.80
α = 0.10, T = 2.00	92.98	93.46	93.15	93.17	96.82	99.80
Noisy Student with α = 0.10, T = 2.00	91.89	92.38	91.87	91.87	96.89	99.82

AUROC, area under the receiver operating characteristic curve; PRAUC, precision-recall area under the curve.

**Figure 6:**
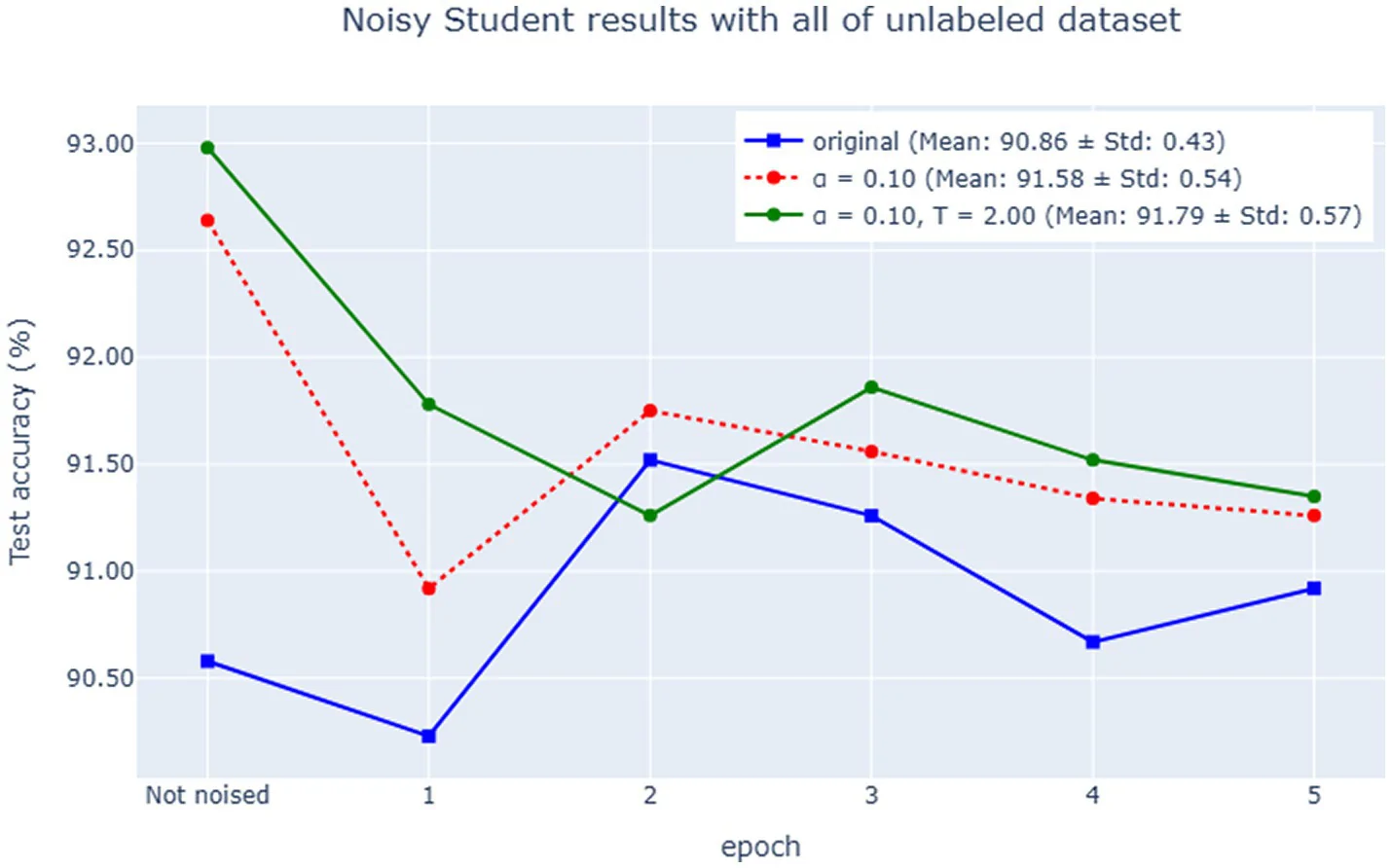
Semi-supervised Noisy Student training algorithm versus various label smoothing and temperature scaling parameters. The x-axis denotes the number of iterations in which the Noisy Student approach was applied to the model, and the y-axis represents the corresponding test accuracy. The three distinct lines illustrate the different scenarios: The blue line represents the model’s performance applying Noisy Student without label smoothing and temperature scaling, the red line represents the model’s performance after using label smoothing (*α* = 0.10) and applying Noisy Student, whereas the green line represents the model’s performance with the best-tuned parameters based on our experiments (*α* = 0.10, *T* = 2.00), combined with the Noisy Student technique.

For the categorical classification model, the fine-tuning label smoothing and temperature scaling relatively improved performance by 2.57% compared with the baseline without any noise-related enhancements (Table 3). For our semi-supervised approach experiment, Figure 6 provides a visual representation of the results obtained by applying the Noisy Student technique to a categorical classification model. Across the Noisy Student self-training iterations, the test accuracy remained stable, with mean ± standard deviation of 90.86 ± 0.43%, 91.58 ± 0.54%, and 91.79 ± 0.57% for the original, label-smoothing (*α* = 0.10), and label-smoothing with temperature-scaling (α = 0.10, T = 2.00) configurations, respectively (Figure 6), indicating stable behaviour across iterations.

To conclude the results of using the two-stage model, soft labelling, temperature scaling, and Noisy Student, we evaluated the end-to-end use case. Regarding the impact of the Noisy Student technique, our findings showed that using all the techniques used in this study improved the model’s accuracy by 10.48%, compared with that of the baseline one-stage model (Table 4). Unless otherwise stated, the class-wise results reported in Section 3.1 and Appendix A correspond to the two-stage model with label smoothing (α = 0.10) and temperature scaling (T = 2.00), which achieved a top-1 accuracy of 92.43%; applying Noisy Student training further improved the end-to-end accuracy to 92.59% (Table 4).

**Table 4:** Model results achieved through the two-stage model classification, employing label smoothing, temperature scaling, and the Noisy Student training approach end-to-end.

Parameter configuration	Accuracy	Precision	Recall	F1-score	AUROC	PRAUC
One-stage model	82.11					
Two-stage model						
Original	91.54	94.39	91.54	92.81	99.02	99.01
α = 0.10, T = 0	92.08	94.85	92.08	93.34	99.02	99.01
α = 0.10, T = 2.00	92.43	95.29	92.43	93.73	99.02	99.01
Noisy Student with α = 0.10, T = 2.00	92.59	95.37	92.59	93.80	99.02	99.01

AUROC, area under the receiver operating characteristic curve; PRAUC, precision-recall area under the curve.

### 3.1 Classification performance analysis and failure cases

The detailed class-wise performance metrics are provided in Appendix A. The classification report, including precision, recall, F1-score, is summarised in Supplementary Table A1, while the confusion matrix is illustrated in Supplementary Figure A1. The proposed framework achieved an overall accuracy of 92.43%, with weighted and macro-average F1-scores of 0.9250 and 0.8988, respectively. Most fruit and vegetable categories achieved high classification performance, with several classes obtaining F1-scores above 0.95, including banana, broccoli, custard apple, jackfruit, and strawberry. However, relatively lower performance was observed for visually similar categories, particularly cantaloupe (F1-score = 0.6400) and musk melon (F1-score = 0.7500), as well as complex food categories such as Somtam (F1-score = 0.7692). These results indicate that the proposed framework can effectively distinguish diverse food categories while remaining challenged by categories with similar visual characteristics or complex appearances.

**Figure 7:**
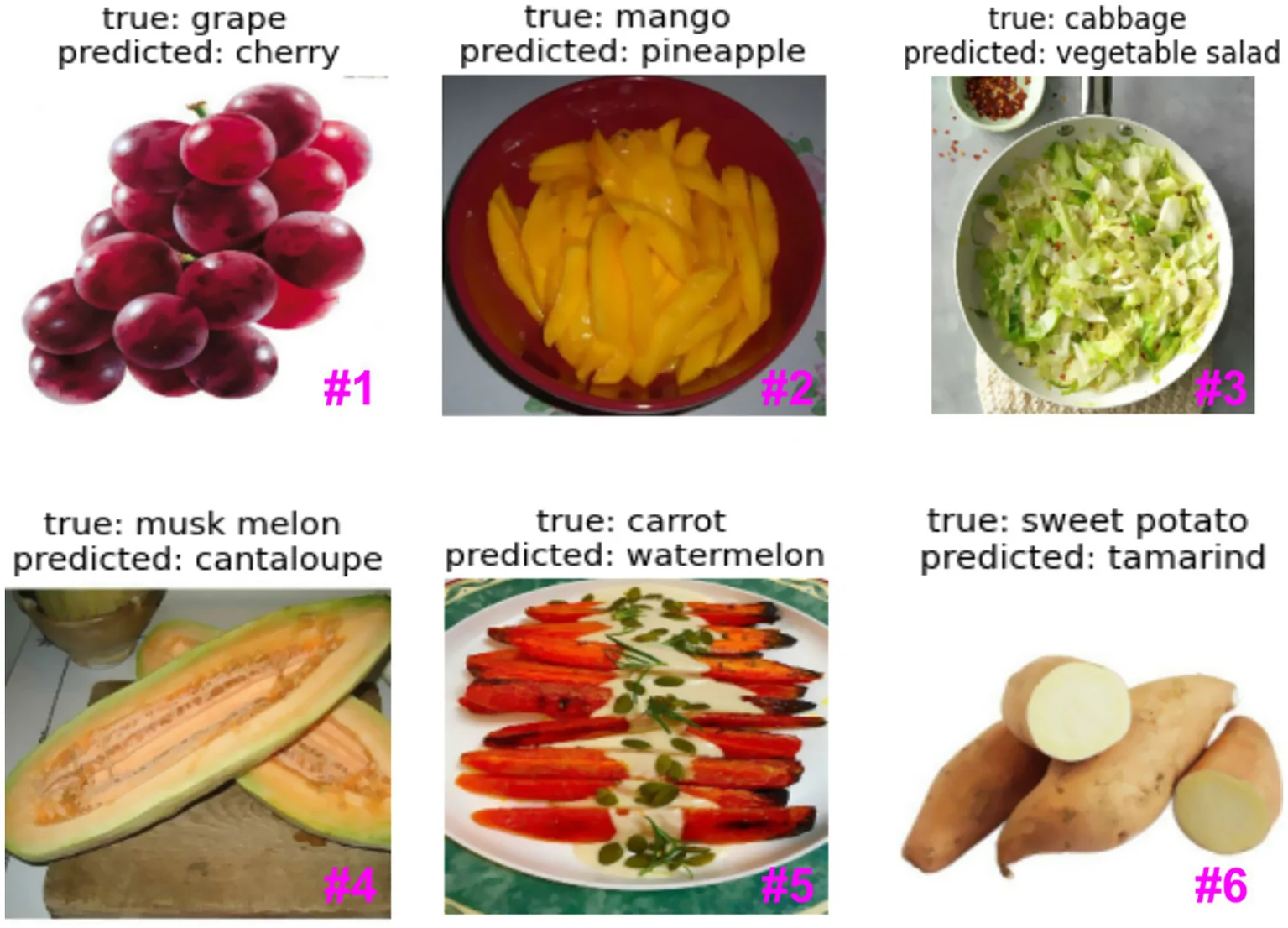
Examples of images with incorrect predictions.

Although the proposed framework achieved high classification performance, several misclassification cases were observed. Figure 7 shows examples of the instances of misprediction by the developed model. In Case 1, an image resembling grapes or cherries was predicted as a cherry by our model because of the high visual similarity in colour and shape between the two categories. In Case 2, there was an erroneous prediction of a sliced mango that resembled a pineapple, likely because the exposed fruit texture and colour resembled those of pineapple. In Case 3, distinguishing between cabbage and vegetable salads based on the input image was challenging due to similarities in appearance and the presence of mixed ingredients. In Cases 4, 5, and 6, the model struggled with more complex images containing variations in background, illumination, object presentation, or image quality, leading to incorrect predictions. These examples indicate that visually similar categories and variations in image acquisition conditions remain challenging for the proposed framework.

### 3.2 Evaluation using a public dataset: Kaggle Fruits-360

To further validate the generalisation capability of the proposed framework, we evaluated it on the public Fruits-360 dataset. We applied the EfficientNetV2S architecture following the procedure outlined in this study and introduced soft labelling and temperature scaling techniques, adjusting parameters *α* and t, respectively. With the proposed parameter adjustment (α = 0.10, T = 2.0), the results demonstrate improvements in accuracy (1.65%), AUROC (2.09%), and the F1 score (1.43%; Table 5).

**Table 5:** Classification performance of our approach on the public dataset Kaggle: Fruits-360.

Parameter configuration	Accuracy	AUROC	F1-score
Original	96.88	96.36	97.77
α = 0.10, T = 0	98.15	98.45	99.17
α = 0, T = 2.0	98.37	98.47	99.57
α = 0.10, T = 2.0	98.53	98.45	99.20

AUROC, area under the receiver operating characteristic curve.

To further position the proposed framework with respect to existing studies, we compared our results with recently published fruit classification methods evaluated on the Fruits-360 dataset. A multi-fused CNN integrating EfficientNet-B0, MobileNetV2, and ResNet50V2 achieved classification accuracies of 99.32 and 97.15% on the 24-class and 131-class Fruits-360 datasets, respectively (43). A VGG16-based model was also reported to achieve competitive performance on a 16-class Fruits-360 dataset (44). In addition, a conventional CNN model achieved an accuracy of 94.35% on the 131-class Fruits-360 dataset (45), while another CNN-based approach reported an accuracy of 98.17% on a 10-class subset of the Fruits-360 dataset (46). Under our experimental setting, the proposed framework achieved an accuracy of 98.53%, an AUROC of 98.45%, and an F1-score of 99.20%, demonstrating competitive performance on the public Fruits-360 benchmark. It should be noted that the compared studies adopted different experimental settings, including different numbers of classes, dataset subsets, network architectures, and training protocols. Therefore, the reported results are intended to provide a general benchmark rather than a direct one-to-one comparison.

## 4 Discussion

In our study, we adopted a two-stage approach comprising a binary and a multi-class model. The findings indicated that integrating label smoothing, temperature scaling, and the Noisy Student technique significantly enhances the performance of deep-learning models for fruit and vegetable image classification. The optimal parameter choices in our study included an alpha (*α*) value of 0.10 for label smoothing, temperature (T) value of 2.00 for temperature scaling, and five iterations of Noisy Student semi-supervised learning. These parameters were highly effective in improving the model performance.

We evaluated the performance of our model using the Fruits-360 dataset available on Kaggle. Notably, the findings confirmed that incorporating soft labelling and temperature scaling enhanced the model’s performance in the image classification of fruits and vegetables, reinforcing its applicability for practical use. This is particularly relevant as higher levels of mobile application engagement have been linked to improved dietary adherence, including significantly greater intakes of fruits and vegetables among high-engagement users compared to low-engagement users (47).

Addressing the inherent limitations of this study is important for providing a comprehensive assessment of our findings and avoiding an undue overestimation of their implications. The first limitation involved the nature of our dataset, which exhibits a notable local bias as it was exclusively sourced from a verified Thai fruit and vegetable website. Consequently, the dataset predominantly comprises Thai fruits and vegetables, potentially rendering the model less effective for classifying fruits and vegetables from regions beyond Thailand. Incorporating a larger number of images covering a broad variety of fruit and vegetable types into a dataset could potentially address this issue and mitigate instances of misclassification.

The second limitation is the limited scope of the parameter grid search conducted in this study. We conducted this experiment using parameters for label smoothing and temperature scaling; nevertheless, the parameter search was conducted within a relatively constrained space. Using a more extensive parameter search with a broader range of values could further improve the model performance. However, caution should be taken to avoid making the model too specialised for this dataset, which can lead to overfitting.

Another limitation is the lack of comprehensive calibration evaluation. Although temperature scaling was applied to improve prediction reliability, metrics such as Expected Calibration Error (ECE) and reliability diagrams were not included. Future work will investigate these analyses to further assess model confidence reliability. In addition, the reported results are based on a single train–test split; repeated cross-validation with confidence intervals was beyond the computational scope of this study and will be considered in future work. Finally, while the model showed the ability to correctly classify fruit and vegetable images, there were instances of misprediction. To address the challenges of ambiguous images, the focus of our efforts in future experiments will be on expanding the dataset with additional images and leveraging confidence values to enhance the model’s performance.

Nevertheless, in this study, we achieved an end-to-end accuracy rate of 94.91% for the classification of 43 different types of fruits and vegetables and three classes of vegetable-heavy dishes, which is sufficiently robust for practical real-world applications.

## 5 Conclusion

In the present study, we developed a robust image classification model for fruits and vegetables that is suitable for resource-constrained mobile applications such as the ThaiSook mHealth application. The model supports automated dietary logging by accurately identifying 46 categories of Southeast Asian fruits, vegetables, and vegetable-heavy dishes. This innovation addresses a key challenge in mHealth dietary monitoring by reducing the burden of manual data entry and enhancing user engagement through timely feedback.

Integrating EfficientNetV2S, combined with semi-supervised learning techniques such as Noisy Student training, label smoothing, and temperature scaling, demonstrated strong performance metrics, achieving an end-to-end top-5 classification accuracy of 94.91%. This performance affirmed the model’s practicality for real-world deployment in mobile settings where computational resources are limited.

Future studies should focus on expanding the dataset to include more diverse food categories beyond Thai cuisine, improving the generalisability of the model to international users. In addition, exploring real-time deployment scenarios and user feedback integration will further enhance the utility and adoption of food image classification systems in mobile health platforms.

## Data Availability

The original contributions presented in the study are included in the article/Supplementary material, further inquiries can be directed to the corresponding author.
