# Supplementary material for: A two-stage deep-learning framework for image classification of 46 southeast Asian fruit and vegetable categories
Source: Front Public Health. 2026 Aug 25;14:1875869. doi: 10.3389/fpubh.2026.1875869 (PMC13550162; doi:10.3389/fpubh.2026.1875869)
Supplement: Supplementary file 1 [file Table_1.DOCX]

**Appendix A.** Detailed Classification Performance Analysis

**Table A1. Class-wise performance metrics of the proposed framework.**

| **Classes** | **Precision** | **Recall** | **F1-Score** | **Support** |
| --- | --- | --- | --- | --- |
| **Apple** | 0.7222 | 0.9630 | 0.8254 | 27 |
| **Bamboo shoot** | 1.0000 | 0.7200 | 0.8372 | 25 |
| **Banana** | 0.9630 | 1.0000 | 0.9811 | 26 |
| **Broccoli** | 1.0000 | 0.9630 | 0.9811 | 27 |
| **Cabbage** | 0.8387 | 0.9630 | 0.8966 | 27 |
| **Cantaloupe** | 0.6400 | 0.6400 | 0.6400 | 25 |
| **Carrot** | 0.8214 | 0.8519 | 0.8364 | 27 |
| **Cherry** | 0.8261 | 0.9615 | 0.9091 | 26 |
| **Cherry tomato** | 0.9231 | 0.9600 | 0.9412 | 25 |
| **Chinese pear** | 1.0000 | 0.9167 | 0.9565 | 24 |
| **Chinese white cabbage** | 0.9200 | 0.9200 | 0.9200 | 25 |
| **Corn** | 0.9310 | 1.0000 | 0.9643 | 27 |
| **Cucumber** | 0.8571 | 0.9600 | 0.9057 | 25 |
| **Custard apple** | 1.0000 | 1.0000 | 1.0000 | 24 |
| **Dragon fruit** | 0.8570 | 0.8400 | 0.8571 | 25 |
| **Durian** | 0.9630 | 0.9630 | 0.9630 | 27 |
| **Grapes** | 0.8519 | 0.9200 | 0.8846 | 25 |
| **Guava** | 0.8214 | 0.9583 | 0.8846 | 24 |
| **Jackfruit** | 1.0000 | 0.9583 | 0.9787 | 24 |
| **Jujube** | 0.7667 | 0.9200 | 0.8364 | 25 |
| **Litchi** | 1.0000 | 0.9167 | 0.9565 | 24 |
| **Longan** | 0.9167 | 0.9167 | 0.9167 | 24 |
| **Longkong** | 0.7586 | 0.8800 | 0.8148 | 25 |
| **Mango** | 0.8462 | 0.8462 | 0.8462 | 26 |
| **Mangosteen** | 0.9259 | 1.0000 | 0.9615 | 25 |
| **Musk melon** | 0.7826 | 0.7200 | 0.7500 | 25 |
| **Orange** | 0.8462 | 0.8800 | 0.8627 | 25 |
| **Others** | 0.9662 | 0.9423 | 0.9541 | 1091 |
| **Pak choi** | 1.0000 | 0.9600 | 0.9796 | 25 |
| **Papaya** | 0.8519 | 0.9583 | 0.9020 | 24 |
| **Pineapple** | 0.9286 | 0.9286 | 0.9286 | 28 |
| **Pomelo** | 0.8333 | 0.8333 | 0.8333 | 24 |
| **Pumpkin** | 0.8400 | 0.8400 | 0.8400 | 25 |
| **Rambutan** | 0.9583 | 0.9200 | 0.9388 | 25 |
| **Rose apple** | 0.9565 | 0.9167 | 0.9362 | 24 |
| **Sapodilla** | 1.0000 | 0.8800 | 0.9362 | 25 |
| **Solanum** | 0.8846 | 0.9583 | 0.9200 | 24 |
| **Somtam** | 0.7143 | 0.8333 | 0.7692 | 24 |
| **Soybean** | 1.0000 | 0.9231 | 0.9600 | 26 |
| **Spinach** | 0.9259 | 0.9615 | 0.9434 | 26 |
| **Star fruit** | 0.8750 | 0.8400 | 0.8571 | 25 |
| **Stir-fried vegetables** | 0.8966 | 0.9286 | 0.9123 | 28 |
| **Strawberry** | 0.9286 | 1.0000 | 0.9630 | 26 |
| **Sweet potato** | 0.9167 | 0.8462 | 0.8800 | 26 |
| **Tamarind** | 0.9565 | 0.8800 | 0.9167 | 25 |
| **Vegetable salad** | 0.8125 | 0.9286 | 0.8667 | 28 |
| **Watermelon** | 0.9565 | 0.8462 | 0.8980 | 26 |
|  | | | | |
| **Accuracy** |  |  | 0.9243 | 2259 |
| **Macro average** | 0.8944 | 0.9077 | 0.8988 | 2259 |
| **Weighted average** | 0.9281 | 0.9243 | 0.9250 | 2259 |

**Figure A1. Confusion matrix of the proposed framework on the test dataset.**
